# Low-Level Laser-Accelerated Peripheral Nerve Regeneration within a Reinforced Nerve Conduit across a Large Gap of the Transected Sciatic Nerve in Rats

**DOI:** 10.1155/2013/175629

**Published:** 2013-05-07

**Authors:** Chiung-Chyi Shen, Yi-Chin Yang, Tsung-Bin Huang, Shiuh-Chuan Chan, Bai-Shuan Liu

**Affiliations:** ^1^Department of Neurosurgery, Taichung Veterans General Hospital, Taichung 40705, Taiwan; ^2^Department of Medicine, National Defense Medical Center, Taipei 114, Taiwan; ^3^Tri-Service General Hospital, National Defense Medical Center, Taipei 114, Taiwan; ^4^Department of Physical Therapy, Hungkuang University, Taichung 43302, Taiwan; ^5^Department of Bioscience Technology, Chang Jung Christian University, Tainan 71101, Taiwan; ^6^Graduate Institute of Pharmaceutical Science and Technology, Central Taiwan University of Science and Technology, Taichung 40601, Taiwan; ^7^Department of Medical Imaging and Radiological Sciences, Central Taiwan University of Science and Technology, Taichung 40601, Taiwan

## Abstract

This study proposed a novel combination of neural regeneration techniques for the repair of damaged peripheral nerves. A biodegradable nerve conduit containing genipin-cross-linked gelatin was annexed using beta-tricalcium phosphate (TCP) ceramic particles (genipin-gelatin-TCP, GGT) to bridge the transection of a 15 mm sciatic nerve in rats. Two trigger points were irradiated transcutaneously using 660 nm of gallium-aluminum arsenide phosphide (GaAlAsP) via laser diodes for 2 min daily over 10 consecutive days. Walking track analysis showed a significant improvement in sciatic functional index (SFI) (*P* < 0.01) and pronounced improvement in the toe spreading ability of rats undergoing laser stimulation. Electrophysiological measurements (peak amplitude and area) illustrated by compound muscle action potential (CMAP) curves demonstrated that laser stimulation significantly improved nerve function and reduced muscular atrophy. Histomorphometric assessments revealed that laser stimulation accelerated nerve regeneration over a larger area of neural tissue, resulting in axons of greater diameter and myelin sheaths of greater thickness than that observed in rats treated with nerve conduits alone. Motor function, electrophysiological reactions, muscular reinnervation, and histomorphometric assessments all demonstrate that the proposed therapy accelerated the repair of transected peripheral nerves bridged using a GGT nerve conduit.

## 1. Introduction

Traumatic injury frequently causes peripheral nerve damage. The most severe form of nerve damage involves complete transection of the nerve, which results in the loss of sensory and motor function at the site of injury. Although a degree of recovery can be expected in most untreated nerve injuries, the process is slow and often incomplete [1]. Despite considerable advances in microsurgical techniques, the functional results of peripheral nerve repair remain largely unsatisfactory. Regrowth of nerves across large gaps is particularly challenging, usually requiring a nerve graft to correctly bridge the proximal and distal nerve stumps. 

At present, nerve autografting is the most common treatment used to repair peripheral nerve defects. However, this recognized “gold standard” technique has a number of inherent disadvantages, such as limited availability of donor tissue [2], secondary deformities, potential differences in tissue structure and size [3], and numbness at donor sites [4]. Although xenografts and allografts have been proposed as alternatives to autografts, the success rate of these techniques remains poor, often resulting in immune rejection [5, 6]. Thus, researchers have invested considerable effort in developing synthetic nerve conduits for the repair of peripheral nerve defects. 

Clinicians have focused on developing more effective methods to promote nerve regeneration, target organ reinnervation, and restore function at the site of injury. Many physical and neurotrophic factors, as well as pharmaceutical drugs, influence nerve regeneration. Physiotherapy commonly involves the use of therapeutic instruments for regenerative purposes [7]. Various forms of external stimulation have been employed to accelerate the process of regeneration, which in turn accelerates functional recovery. Such techniques include electrical [8], ultrasound [9], and low-level laser (LLL) stimuli. The effectiveness of LLL therapy in treating peripheral nerve damage has been widely demonstrated. 

Very few studies have employed tubulation in combination with diode laser therapy to repair nerve lesions. Furthermore, very few researchers have investigated the influence of LLL therapy on neural regeneration in a biodegradable nerve conduit. This study used a previously developed biodegradable composite containing genipin-cross-linked gelatin annexed with *β*-tricalcium phosphate ceramic particles (GGT) as a nerve conduit [10, 11] and trigger point therapy to treat peripheral nerve injuries in rats. The multichannel LLL system was developed from Chinese traditional acupuncture and laser biostimulation theory. In the present study, a GGT nerve conduit was used to bridge a 15 mm critical defect gap in the sciatic nerve of a rat. 660 nm gallium-aluminum-arsenide phosphide (GaAlAsP) LLL treatments were subsequently applied transcutaneously to the transected nerve for 2 min daily over 10 consecutive days. The effects of LLL therapy on peripheral nerve restoration and regeneration were systematically investigated throughout the study period. This paper presents our general postoperation observations, nerve function analysis, electrophysiological results, gastrocnemius muscular atrophy evaluation, and histomorphometric assessments. We believe that this research will be of clinical value in the future treatment of nerve injuries.

## 2. Materials and Methods

### 2.1. Materials

Type A gelatin powder (300 Bloom, Sigma Chemical Co., St. Louis, MO, USA) was extracted and purified from porcine skin with an average molecular weight of about 50,000–100,000 Dalton measured with SDS-PAGE. Genipin was obtained from Challenge Bioproducts Co. (Taichung, Taiwan). The beta-tricalcium phosphate Ca_3_(PO4)_2_ ceramic particles were purchased from Sigma-Aldrich (Germany).

### 2.2. Nerve Conduit Fabrication

A homogeneous 18% gelatin solution (Bloom number 300, Sigma, St. Louis, MO, USA) was prepared in a water bath at 60°C. The gelatin solution was then mixed with weight ratios (gelatin/TCP = 1/2) of *β*-tricalcium phosphate (*β*-TCP, Fluka, Alzenau, Germany) ceramic particles to obtain the final gelatin-TCP (GT) mixture. A silicone rubber tube (Helix Medical, Inc., Carpinteria, CA, USA) was used was used as a template (an inner mandrel), by being vertically dipped into the mixture at a constant speed where it remained for 1 min. The mandrel was then slowly withdrawn and air dried for 5 min. The mandrel was rotated horizontally to reduce variations in the wall thickness along the axis of the tube. The coating process was performed in three steps to obtain a GT tube. The GT-coated mandrel was then immersed in a 1% (w/w) genipin solution (Sigma Chemical Co., St. Louis, MO, USA) for 48 h to allow time for sufficient cross-linking reactions within the gelatin. The genipin-cross-linked GT tubes were labeled GGT nerve conduit. The GGT-coated mandrel was rinsed twice with distilled water and dried in a freezedryer before the GGT conduits were removed from the silicone rubber template. Finally, the hollow GGT conduits were sterilized using 25 kGy of ^60^Co gamma ray irradiation (China Biotec Co., Taichung, Taiwan) and stored in a desiccator at room temperature.

### 2.3. Morphological Observation of GGT Nerve Conduits

We examined both the macroscopic appearance and microscopic structure of the GGT nerve conduits. The surface morphology was characterized in cross-sections and longitudinal sections using a scanning electron microscope (SEM, model S-3000N, Hitachi, Japan) at an accelerating voltage of 5 kV. Prior to morphological observation, the specimens were mounted on metal tabs using carbon adhesive tape and coated with gold using a sputter coater (model E-1010 Ion Sputter, Hitachi, Japan) in an argon atmosphere. The inner diameter and wall thickness of the GGT nerve conduits were measured directly from the SEM images.

### 2.4. Experimental Groups and Surgical Procedure

Twenty adult male Sprague-Dawley rats, weighing approximately 250–350 g, were provided by the National Laboratory Animal Center, National Applied Research Laboratories (NARL). The animalswere randomly divided into two groups. In the control group (GGT, *n* = 10), animals were sham-irradiated controls. In the laser-treated group (GGT/LS, *n* = 10), animals received a treatment of low-level laser stimulation. Each group of animals was left in place for twelve weeks, whereupon the grafts were harvested together with the proximal and distal nerve stumps. The animals were anesthetized using an inhalational anesthetic (VMS, Matrx, NY, USA). Following the incision of the skin, the fascia and muscle groups were separated using blunt dissection, and the left sciatic nerve was severed into proximal and distal segments. The proximal stump was then secured using a single 10-0 prolene suture (Johnson-Johnson, NJ, USA) through the epineurium and the outer wall of the GGT nerve conduits (17 mm in length). The distal stump was secured similarly into the other end of the chamber. Both the proximal and the distal stumps were secured to a depth of 1 mm into the chamber, leaving a 15 mm critical gap between the stumps. For all of the animals, the right contralateral sciatic nerve served as a positive control. The muscle layer was reapproximated using 4-0 chromic gut sutures, and the skin was closed using 2-0 silk sutures. Following surgery, the rats were housed separately under temperature- and humidity-controlled conditions with a 12 h light/12 h dark cycle and free access to food and water. Prior to the beginning of the study, the protocol was approved by the Ethics Committee for Animal Experiments of Central Taiwan University of Science and Technology, Taichung, Taiwan.

### 2.5. Trigger Point Laser Therapy

**Figure 1:**
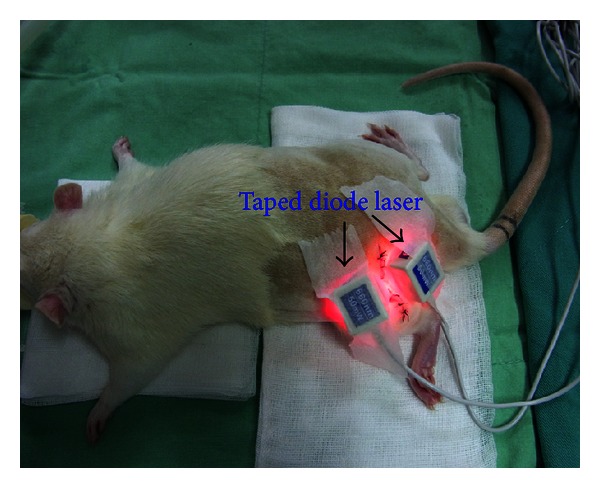
Rats were subjected to treatment of low-level laser irradiation.

The diode laser (Aculas-AM-100A, Konftec Co., Taipei, Taiwan) used in this experiment is a multichannel LLL system designed for trigger point therapy. This device has five gallium-aluminum-arsenide phosphide (GaAlAsP) laser diodes directly taped to the trigger point, with no risk of laser leakage. When set to continuous mode, the laser emits a wavelength of 660 nm at a power of 50 mW with a beam area of 0.1 cm^2^. Two trigger points along the surgical incision of the sciatic nerve were irradiated transcutaneously for 2 min per exposure; a distance of 2 cm separated the trigger sites.  Figure 1 presents the GGT/LS group of rats treated with LLL therapy. Laser therapy began the day following the operation; one treatment per day was applied for 10 consecutive days. The animals were handled gently; laser biostimulation did not produce any painful sensation or distress to the animals. The animals in the control group were subjected to the same procedure, but with the laser switched off.

### 2.6. Nerve Function Analysis

Nerve function recovery was assessed by calculating the sciatic functional index (SFI) as described by Bain et al. [12]. At predetermined times, the animals were evaluated for the recovery of nerve function using walking track analysis. Following the 5 minute initial gait training, the hind paws of the rats were pressed onto an inkpad whereupon the rats were allowed to walk the length of a track leaving their hind footprints on the paper. Three measurements were taken from the footprints: (1) the print length (PL, distance from the heel to the third toe); (2) the toe spread (TS, distance from the first to the fifth toe); (3) the intermediate toe spread (ITS, distance from the second to the fourth toe). All three measurements were taken from both experimental (E) and normal (N) sides. Three factors that comprised the sciatic function index were calculated as follows: (1) print length factor (PLF) = (EPL − NPL)/NPL; (2) toe spread factor (TSF) = (ETS − NTS)/NTS; (3) intermediary toe spread factor (ITF) = (EIT − NIT)/NIT. Using these data, the SFI, which indicates the differences between the injured and the intact contralateral paw, was calculated according to the following equation [12]:

(1) $$\begin{array}{c} \text{SFI}=-38.3\times \text{PLF}+109.5\times \text{TSF}+13.3\times \text{ITF}-8.8. \end{array}$$

### 2.7. Electrophysiological Study

All animals that had undergone apparent nerve regeneration were reanesthetized, and the left sciatic nerve was carefully exposed. Electrophysiological analysis was carried out by stimulating the proximal end of the graft with a bipolar stimulating electrode connected to a DC electrical stimulator (ML856 Power Lab 26T, AD Instruments, Sydney, Australia), with the ground electrode positioned on the tail of the rat. The nerve stimulation parameters included the following: 4 Hz pulses, 200 mV in strength, 0.2-ms pulse duration, to elicit compound evoked muscle action potential (CMAP). Muscle contractions were recorded from gastrocnemius muscles with microneedle electrodes linked to computer software (LabChart 7, AD Instrument, Sydney, Australia); the ground electrode was also placed in surrounding muscle tissues to remove conduction of stimulation through muscle tissues. The amplitude and the area under the CMAP curves from the baseline to the maximal peak were calculated. Normal CMAP was measured on the contralateral nonoperated side. The recovery index was calculated as the peak amplitude and the area under the CMAP curves of regenerated side divided by those of contralateral nonoperated side.

### 2.8. Muscle Mass Preservation

Using an operating microscope, both the left (operated side) and right (nonoperated side) gastrocnemius muscles were carefully cleaned and resected, dividing the tendinous origin and insertion from the bone. Muscles were weighed following harvesting. The decline in muscle mass was quantified by weighing the excised gastrocnemius muscle and calculating the ratio of muscle weight between the two legs (referred as L/R), using the following formula: L/R = weight operative muscle/weight normal muscle. A reduction in atrophy was observed as the L/R ratio approached 1.

### 2.9. Histological and Osmium Tetroxide Assessment

After twelve weeks, the rats were perfused transcardially with ice-cold 10% phosphate buffered formalin, and the implanted GGT nerve conduits were removed. Macrographs of GGT nerve conduits including fibrous tissue encapsulation and the appearance of degradation were analyzed. The implants were reexposed and carefully isolated from the surrounding tissue. The nerve segments were then cut 4 mm proximal to the implant and 4 mm distal to the tibial and peroneal nerve branches. Sciatic nerve sections were taken from the middle regions of the implant. Some of the nerve grafts were immediately fixed in 4% paraformaldehyde for 24 h and then washed in 0.1 M phosphate-buffered saline (PBS). After fixation, the segments were dehydrated in a graded ethanol series and finally embedded in spurs. The embedded nerves were then cut longitudinally to a thickness of 4 *μ*m using a microtome with a dry glass knife and stained with basic haematoxylin and eosin (H&E). The sections were observed using light microscopy (Nikon's Eclipse E600, CA, USA). Furthermore, some segments were then fixed in 1% osmium tetroxide (Sigma, O5500, St. Louis, MO, USA), washed in graded alcohol, and embedded in Epon resin. Finally the nerve samples were cut transversely to a thickness of 4 *μ*m. With the observer blinded to the identity of the groups, sections of the regenerating bridge (approximately at its center) were photographed using a high-power light microscope. An image analyzer system (Image-Pro Lite, Media Cybernetics, Silver Spring, MD) coupled to the microscope was used to count the number of nerve fibers and calculate the area of medial nerves, the diameter of nerve fiber and axons, and the thickness of the myelin sheaths in each section of the nerve. Between 30% and 50% of the nerve section area was randomly selected from each nerve specimen for observation at a magnification of 400x. Using Image-Pro Plus software, photographs were taken of each section to be used in the final quantitative analysis.

### 2.10. Immunohistochemical Staining

Some of the nerve grafts were freshly frozen in Tissue-Tek OCT medium and sectioned. The frozen sections were blocked with 5% skimmed milk in PBS for 30 min at room temperature. The sections (10 *μ*m thick) were incubated with primary antibodies at 4°C overnight. The primary antibody against rabbit polyclonal antibody S-100 (1 : 40, Serotec, Kidlington, UK) was used. The sections were then rinsed with PBS and incubated for 20 min at room temperature with *Super PicTure* Polymer Detection Kit (Invitrogen, Cat. no. 87-8963, Carlsbad, CA, USA). Finally, the sections were visualized using color development with 3,3′-diaminobenzidine (DAB) for 3–8 min and counterstained with hematoxylin. The immunostained sections were mounted with Clearmount (Invitrogen Cat. no. 00-8110, Carlsbad, CA, USA) and examined using a light microscopy (Nikon Eclipse E 600; Nikon, Thornwood, NY, USA).

### 2.11. Statistical Analysis

This study proposed a linear mixed-effects model [13] to facilitate a comparison of results. The proposed model considers individuals as random effects and groups as fixed effects, employing temporal variations and interactions among them for the calculation of mean, standard deviation, and the coefficient of variation. All numerical data are presented as mean ± standard deviation. The differences among the results obtained under various testing conditions were evaluated using two-sample *t*-tests without equal variance. The level of statistical significance was set at *P* < 0.05.

## 3. Results

### 3.1. Appearance and Morphology of GGT Nerve Conduits

**Figure 2:**
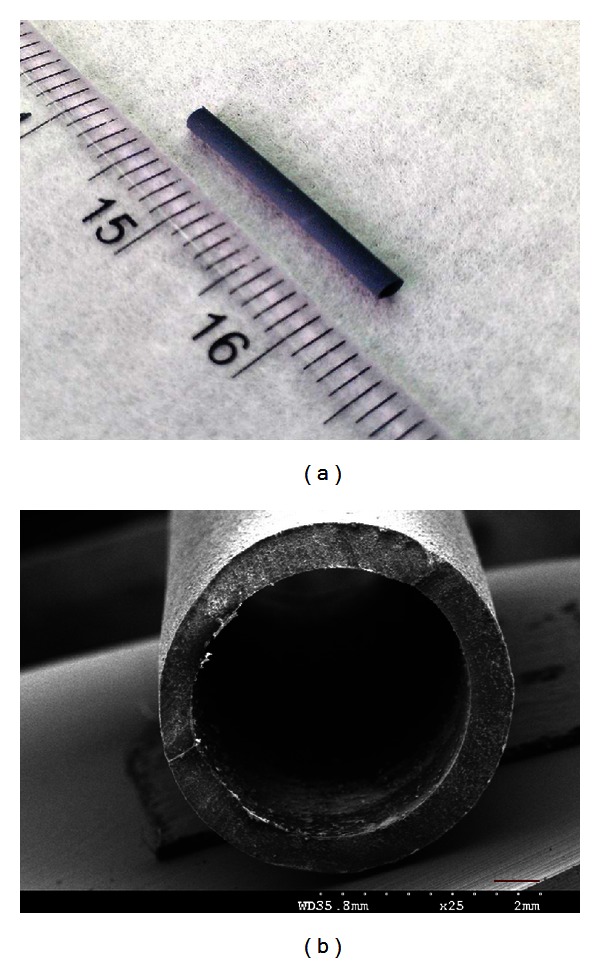
Appearance and morphology of the GGT nerve conduit. (a) Macrograph. (b) SEM micrograph. The ruler is metric and the scale bar representing 400 *μ*m.

Figure 2 presents a macrograph and scanning electron microscope (SEM) micrograph of the GGT nerve conduit. The GGT nerve conduit was a hollow tube with a uniformly opaque, dark bluish appearance. It had an inner diameter of 2.45 ± 0.03 mm and a wall thickness of 0.42 ± 0.02 mm. SEM cross-sectional and longitudinal images of the GGT nerve conduit show that it was concentric with a rough, compact outer wall and a smooth inner lumen.

### 3.2. General Observations after Operation

**Figure 3:**
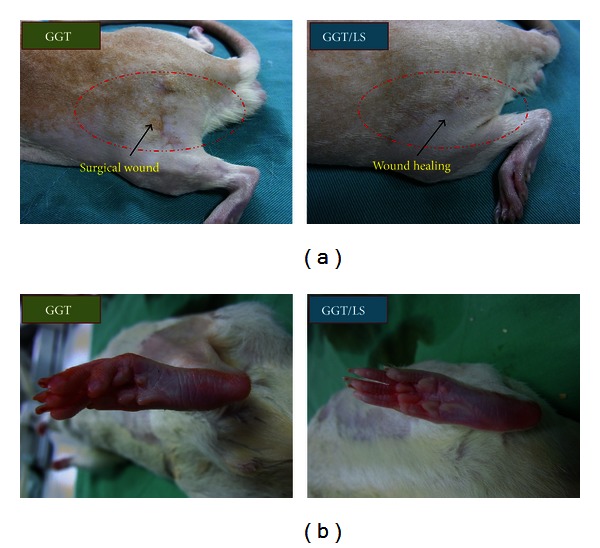
Macrographs of (a) incised skin and (b) the hind foot of rats in the experiment. The left leg was operated.

The animals in this study showed no sign of infection after anesthetic and operative procedures. Moreover, no signs of discomfort were observed throughout the twelve-week evaluation period. All animals in both the GGT and GGT/LS groups survived the experimental period and displayed normal eating and drinking behavior throughout. The percentage of weight change in the laser-treated group was significantly higher than in the sham-irradiated group at 6 weeks after operation (*P* < 0.05) (data not shown in this study).  Figure 3 presents macrographs of the incised skin and the operated hind foot of rats in the experiment. After shaving the hair at the suture site five weeks after operation, we observed that the wound in the GGT/LS group had healed more rapidly than that of the GGT group (Figure 3(a)). Following the creation of severe nerve lesions, the rats tended to gnaw the insensate toes of a partial phantom limb. However, six weeks after surgery, automutilation was not observed in either the GGT or GGT/LS group. Furthermore, none of the animals developed digit flexion contracture within the period of observation (Figure 3(b)).

### 3.3. Walking Track Analysis

**Figure 4:**
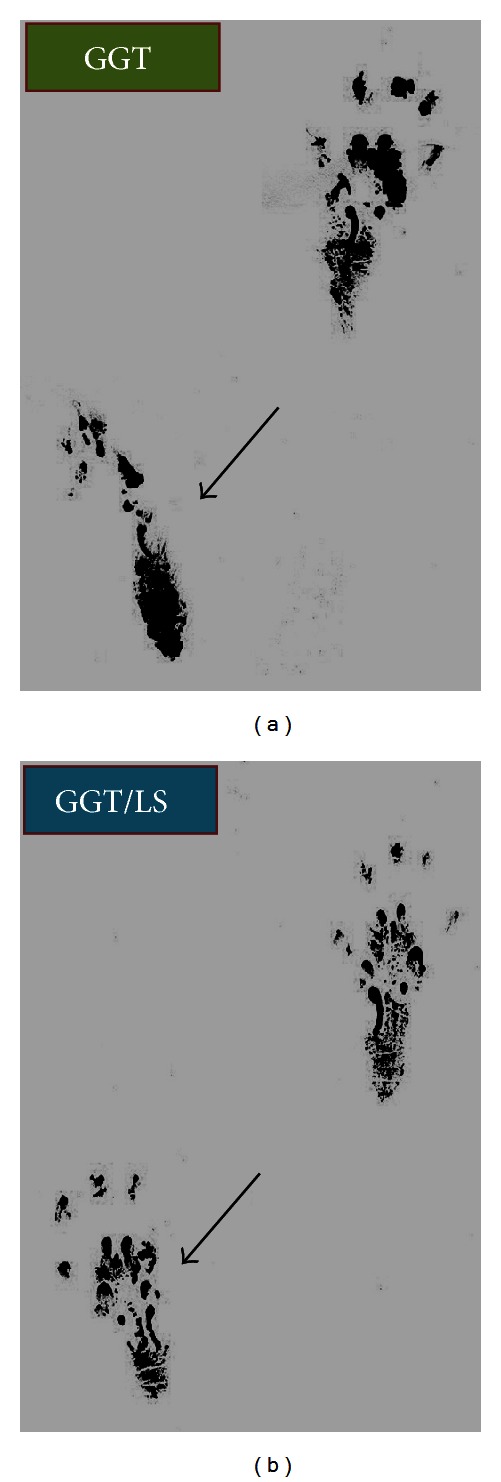
Footprint stamps in walking track analysis at twelve weeks following GGT nerve conduit implantation in the (a) sham-irradiated and (b) laser-treated groups (black arrows denote the injured paw).

Table 1 illustrates the recovery of sciatic nerve function in the twelve weeks following the operation. In the first postoperative evaluation (one week), the footprints of all animals were longer and narrower on the operated side than the normal side, because the animals used both the heel and the shrunken toes when walking and standing. The SFI increased considerably over time; a similar trend of postoperative improvement was observed in both groups. The improvement in SFI values indicates that some regenerated axons passed through the implant and reached the target organ. Walking track analysis shows a significantly higher SFI value in the GGT/LS group than in the GGT group at 6, 8, 10, and 12 weeks (*P* < 0.01), indicating that animals in the sham-irradiated group were experiencing slower motor recovery than the animals that received LLL irradiation treatment.  Figure 4 presents the footprints from walking track analysis twelve weeks after the implantation of GGT nerve conduits. These grayscale images of footprints of GGT/LS group animals show a greater improvement in toe spreading ability. The footprints themselves were also shorter and wider than those of the GGT group. In summary, the results of sciatic nerve function recovery indicate the superiority of the motor function of rats in the GGT/LS group.

### 3.4. *In Vivo* Electrophysiological Analysis

**Figure 5:**
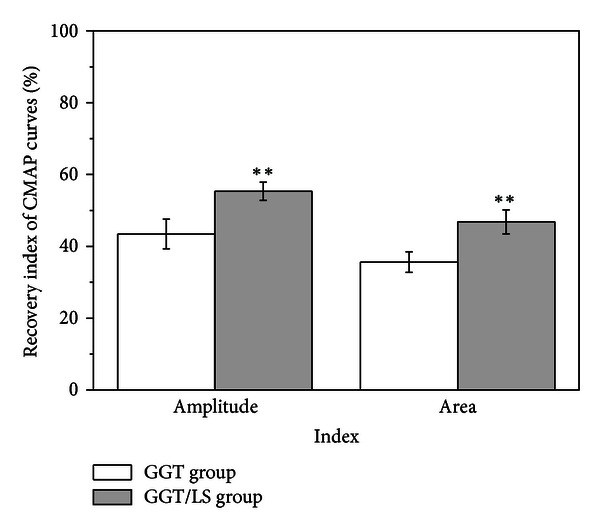
Analysis of electrophysiological data recording the peak amplitude and the area of the CMAP curves twelve weeks after operation. **Significance (*P* = 0.001 for amplitude and *P* = 0.002 for area, *P* < 0.01) greater than the GGT group.

To quantify the functional recovery of regenerated nerves, electrophysiologic analysis was performed at twelve weeks after operation. This investigation assessed the response of the gastrocnemius muscle in the hind limb to electrical stimulation at the proximal end of the grafts.  Figure 5 presents the peak amplitude and area under the CMAP curves from the experimental limb, normalized to values from the contralateral control. At twelve weeks after implantation, the mean recovery index (peak amplitude) shows that the GGT/LS group (55.34 ± 2.52) had significantly superior functional recovery than the GGT group (43.45 ± 4.13) (*P* < 0.01). The mean recovery index (area under the CMAP curve) in the GGT/LS group (46.86 ± 3.34) was also significantly higher than that in the GGT group (35.62 ± 2.87) (*P* < 0.01).

### 3.5. Biocompatibility and Sciatic Nerve Regeneration

**Figure 6:**
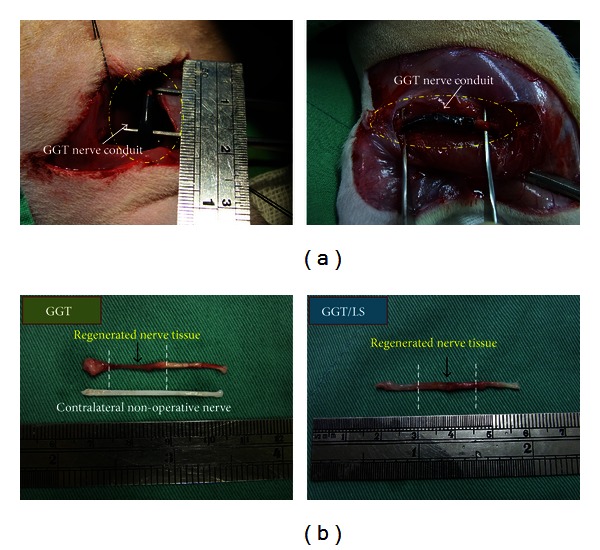
(a) Intraoperative photographs of the GGT nerve conduit immediately (left) and twelve weeks (right) after implantation. (b) Photographs of the sciatic nerve of an adult rat twelve weeks after GGT nerve conduit reconstruction; completely regenerated nerve continuity was observed (shown in black arrows).

Figure 6(a) presents macrographs of the sciatic nerve twelve weeks after grafting the GGT nerve conduit. The results indicated no gross inflammatory reaction of peripheral nerve tissues at the implantation site. These findings further demonstrate that the implants did not lead to infection and were biocompatible with the peripheral nerve tissues in both groups. We did not observe a dislocation in the implants or neuroma formation at the proximal or distal coaptation site in any of the rats. Moreover the boundaries between nerve tissue and conduit were barely distinguishable in either group. During dissection, the implants were encapsulated in very thin fibrous tissue. This indicates that the host tissue tolerated the GGT nerve conduits and the conduits elicited only a mild foreign body reaction. Visual inspection after surgery revealed that the GGT nerve conduits maintained their round shape and uniform diameter along the entire length of the lumen.  Figure 6(b) presents macrographs of the retrieved specimen of GGT nerve conduit at twelve weeks after operation. After trimming the fibrous tissue and cutting the wall of the implants, we discovered that the nerve tissue had reconnected and bridged the lesion. A white tubular substance was observed throughout the lumen of the implants. Morphologically, the previously transected nerves in both the GGT and GGT/LS groups showed strong reconnection. We observed the growth of regenerated tissue passing through the length of the GGT nerve conduit as well as axonal sprouting through the area of the conduit which connected to the distal portion of the nerve. Furthermore, the GGT/LS group showed more extensive regenerated tissue than the GGT group. These results demonstrate that the GGT nerve conduits acted as *in vivo* nerve guides until nerve reconnection was established, whereupon they were resorbed. Our findings also suggest that LLL therapy promoted nerve regeneration and shortened recovery time.

### 3.6. Gastrocnemius Muscular Atrophy Evaluation

**Figure 7:**
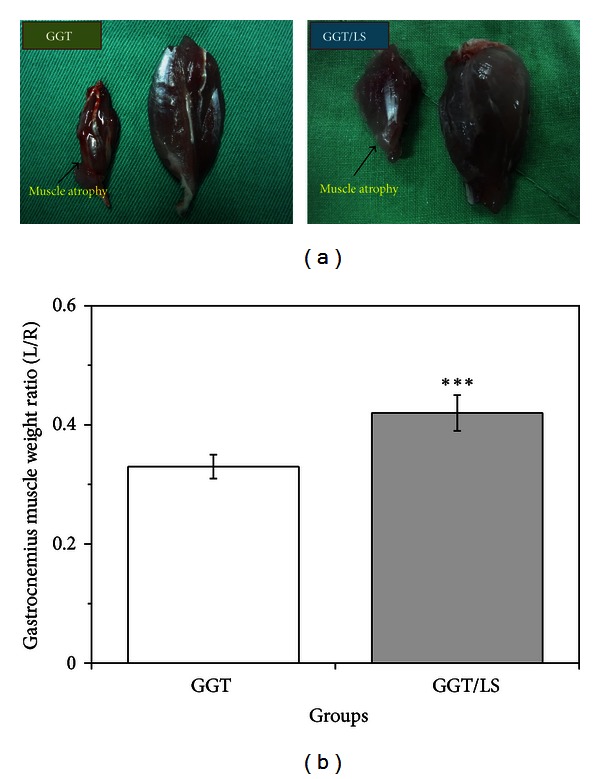
(a) Photographs of muscle atrophy twelve weeks following total axotomy (experimental muscles are on the left, and contralateral muscles are on the right). (b) Graph of the mean gastrocnemius muscle weight ratios (left/right, L/R). ***Significance (*P* < 0.001) greater than the GGT group.

Figure 7 illustrates atrophy in the gastrocnemius muscle twelve weeks after total axotomy. A total transection of the sciatic nerve reduces neural innervations to the gastrocnemius muscle, which leads to a decrease in muscle weight (Figure 7(a)). A significantly lower L/R ratio was found in the GGT group than in the GGT/LS group (GGT: 0.33 ± 0.02 versus GGT/LS: 0.42 ± 0.03) (*P* < 0.001) (Figure 7(b)). The significantly higher muscle weight of laser-treated rats indicates that GGT/LS animals enhanced neural recovery and possessed a protective effect against muscle atrophy.

### 3.7. Histological and Immunohistochemical Analysis

**Figure 8:**
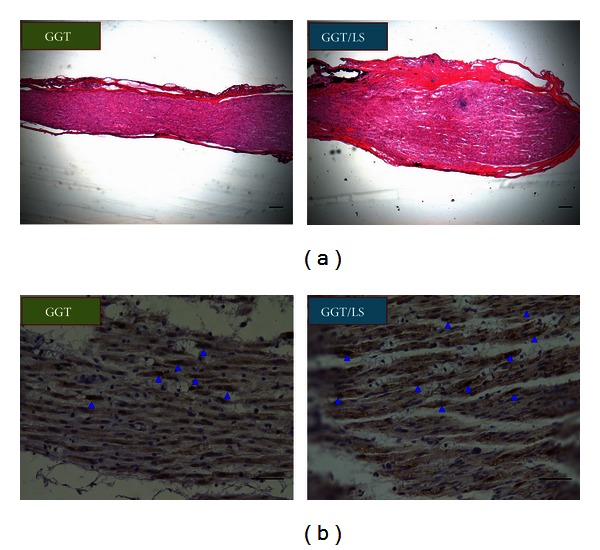
Longitudinal sections with (a) H&E staining and (b) S-100 staining of sciatic nerves from the medial segment of the regenerated nerve tissue twelve weeks after operation in the GGT and GGT/LS groups. Scale bar represents 200 *μ*m. Arrow heads indicate S-100 immunostained Schwann cells (blue).

Figure 8 presents longitudinal sections of the regenerated nerve tissue in the GGT nerve conduit of both GGT and GGT/LS groups twelve weeks after operation. Longitudinal sections in the injury site of the sciatic nerve were analyzed using hematoxylin (H&E) staining. Partial nerve regeneration was observed in the GGT group; however, the regenerated nerve tissue in the GGT/LS group was thicker and presented structures that appeared to be linearly ordered within the resected nerves. Furthermore, the GGT/LS group showed more compact and complete histomorphological regeneration of nerve tissue, compared with the GGT group (Figure 8(a)). Immunohistochemical results indicate that the GGT group expressed a small amount of mature S-100 proteins; however, the GGT/LS group expressed a higher quantity of S-100 proteins (Figure 8(b)). 

### 3.8. Remyelination of Regenerated Nerves

**Figure 9:**
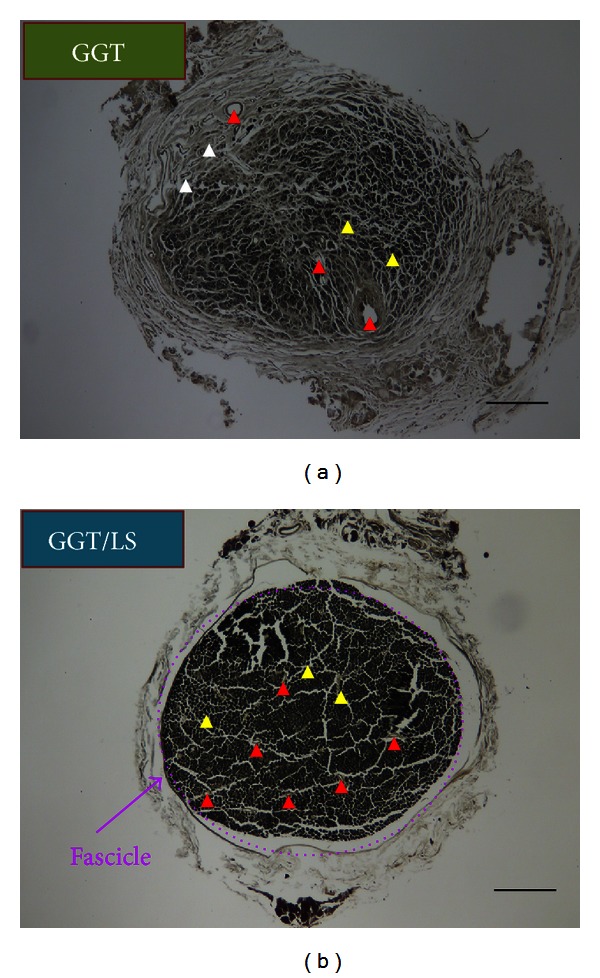
Transverse sections with osmium tetroxide of sciatic nerves from the medial segment of the regenerated nerve tissue twelve weeks after operation in the (a) GGT and (b) GGT/LS groups. Scale bars represent 100 *μ*m. Arrow heads indicate myelinated nerve fibers (yellow), unmyelinated nerve fibers (white), and blood vessels (red).

After twelve weeks, sciatic nerve sections were taken from the middle regions of the implant. The morphological parameters of nerve regeneration in the transverse sections were then evaluated with osmium tetroxide stain.  Figure 9 presents light micrographs of transverse sections of the regenerated nerve tissue at twelve weeks after implantation. Examination revealed a clear qualitative difference between the GGT group and the GGT/LS group. The GGT group presented a relatively low density of myelinated nerve fibers (Figure 9(a)). In contrast, the GGT/LS group presented a higher density of myelinated nerve fibers in the medial portion of the graft (Figure 9(b)). In the GGT group, the untreated nerves were populated mainly by unmyelinated nerve fibers immersed in a collagen fibril rich matrix. Many of these nerve fibers were in the beginning stages of the myelination process. In contrast, myelinated nerve fibers in the regenerated nerve tissue of the laser-treated rats were distributed among many large fascicles. Many well-myelinated axons with large diameters and evenly distributed intraneural blood vessels were observed in the medial portion of the graft. This indicates that angiogenesis had occurred during the nerve regeneration process. Qualitatively, sections from the GGT/LS group exhibited numerous large, well-myelinated axons and histomorphological compaction. Both conditions were found to be less developed in the GGT group.  Table 2 shows a quantitative analysis comparing regenerated nerve tissue of the GGT and GGT/LS groups. All morphological data obtained from the regenerated nerve tissue in the GGT/LS group was significantly greater than that of the GGT group (*P* < 0.01). The quantitative results indicate superior nerve regeneration of the laser-treated group compared with the sham-irradiated group. These results suggest that laser phototherapy accelerates and improves the regeneration of injured peripheral nerves in rats.

## 4. Discussion

The suitability of nerve guide conduits depends a number of factors: partial permeability, which prevents fibrous scar tissue from invading the growing nerve while facilitating the permeation of nutrients and oxygen; revascularization to improve the supply of nutrients; immunological inertness with the surrounding tissue; biodegradability to prevent chronic inflammatory response or pain resulting from nerve compression; the ease of surgical insertion [14]. A substitute for the peripheral nerve requires adequate mechanical durability to withstand handling and suturing during surgery. It must also be flexible enough not to crack or break when the patient moves and to support axonal growth throughout the period of tissue formation [15]. In our previous study [10, 11], we developed a novel biodegradable GGT nerve, composed of genipin-cross-linked gelatin annexed with *β*-TCP ceramic particles to enhance mechanical strength. The proposed GGT nerve conduit is dark bluish in color due to the reaction between the ester groups in genipin and the amino groups in gelatin. The hollow inner lumen of this conduit helps guide the growth of nerve fibers. Moreover, the rough and compact outer wall prevents infiltration by fibrous tissue, the invasion of which can inhibit axonal extension. Furthermore, the GGT nerve conduit retains lumen growth-promoting molecules released by nerve stumps. Our previous study confirmed that TCP ceramic particles structurally reinforced the genipin-cross-linked gelatin structure. The enhanced mechanical properties of the nerve conduit allow it to resist muscular contractions and maintain a support structure with enough space and stability to facilitate nerve regeneration. Since the collapse of an unfilled circular conduit would be a major obstacle to nerve regeneration, combining the gelatin tube with TCP ceramic particles while maintaining its moldability could improve nerve regeneration techniques. 

Clinical and experimental studies have provided evidence that lasers can increase nerve function, reduce the formation of wounds, increase the metabolic activity of neurons, and enhance myelin production [16]. The noninvasive nature of laser phototherapy enables treatment without surgical intervention. This study applied LLL as a noninvasive stimulus to investigate the feasibility of employing lasers to enhance nerve regeneration through a GGT nerve conduit. This study adopted a GaAlAsP diode laser (660 nm) for trigger point therapy due to the low intensity of the laser and because this wavelength is commonly employed in clinical practice. It was developed from Chinese traditional acupuncture and laser biostimulation theory. The biological action of laser radiation in the visible range of the electromagnetic spectrum, and also in clinical application, is based on three reactions: (1) photodynamic action on membranes, which is accompanied by an increase in intracellular calcium and cell stimulation; (2) photoreactivation of Cu-Zn superoxide dismutase (SOD); (3) photolysis of metal complexes formed with nitric oxide following the release of this vasodilator. It has been postulated that these three reactions underlie the indirect bactericidal, regenerative, and vasodilatory outcomes of laser radiation [17]. The therapeutic effects of LLL treatment also have been shown to accelerate the healing of wounds [18] and attenuate pain [19]. We first evaluated changes in weight, the degree to which the incision wound had healed, and the operated hind foot in experimental rats. Our results reveal that LLL irradiation prompted a greater increase in weight (data not shown), as well as more rapid wound healing than that found in sham-irradiated rats. Furthermore, at six weeks after surgery, automutilation was not observed in either the GGT or GGT/LS group.

Evaluation of functional gait is a noninvasive means to assess specific aspects of nerve recovery. The images of footprints were analyzed using the sciatic functional index (SFI), which is a quantitative method considered by many researchers to be reliable and reproducible. We used SFI to assess the results of the repeated ANOVA measurement, due to the high correlation between motor function recovery and morphological/morphometric regeneration of peripheral nerves after injury [20]. In this study, the SFI in the GGT/LS group improved steadily over time, reaching a significantly higher peak level than that of the GGT group at twelve weeks after surgery. Many materials (both natural and synthetic) have demonstrated favorable histomorphometric results when used in peripheral nerve regeneration therapy. However, the appearance of sprouts from the proximal stump at the distal nerve stump (as we observed histologically) does not necessarily indicate recovery of nerve function. Therefore, we measured compound muscle action potential (CMAP) to investigate the reinnervation of the distal motor units. The amplitude of CMAP can be used to estimate the number of functioning motor units (i.e., motor axons and innervated muscle fibers) [21]. Corresponding segments of the transected sciatic nerves (bridged with GGT nerve conduits) in experimental group rats were exposed to LLL irradiation for 10 consecutive days starting the day after surgery. The recorded peak amplitude and area under the CMAP curves in these animals were significantly higher than those of the non-LLL irradiation group twelve weeks after operation (*P* < 0.01). Thus, the laser-treated rats demonstrated superior functionality to that of the sham-irradiated rats. Our observations show that the use of GaAlAsP (660 nm) diode laser in trigger point therapy produced greater improvements in nerve functionality than the GGT treatment alone. It should be noted that neural tissue was located in superficial layers, which may have prompted a better response to this wavelength. Additionally, the use of laser therapy within 24 hours of injury may have reduced the immediate loss of function, confirming the claims made by Dahlin [22]. This suggests that lasers could benefit the process of nerve regeneration beyond the duration considered in this study.

Macroscopic observation of the implantation site found no signs of gross inflammatory reaction in the peripheral nerve tissue. However, the regenerated nerve cables were surrounded by fibrous connective tissue after the GGT nerve conduit gradually degraded. As previously reported, foreign-body reactions are relatively mild in the case of slowly degrading biomaterials. (Reactions tend to be more severe for quickly degrading biomaterials.) The slow degradation of the GGT nerve conduits provided adequate space for neural regeneration before the formation of fibrous tissue could impair the maturation of regenerated nerve tissue. The thin diameter of the fibrous tissue layer surrounding the GGT nerve conduits and the minimal inflammatory reaction they elicited indicate that these conduits are biocompatible with host tissue. Macroscopic observations also found no serious swelling or deformation of the GGT nerve conduits throughout the experimental process. The stability of GGT nerve conduit dimensions may be due to their cross-linked structure or the mechanical strength of the bioactive ceramic TCP particles [10, 11], which likely played a critical role in the success of nerve regeneration. The mid-tube thickness of the intratubular regenerated nerve fibers in the GGT/LS group exceeded that of the GGT group during the twelve-week evaluation period. Relative muscle weight ratio, defined as the ratio of the gastrocnemius muscle weight from the operated side (left) to that of the nonoperated side (right) side (L/R), is an indirect method of estimating sciatic nerve regeneration. In this study, the GGT/LS group demonstrated a significant improvement in wet muscle weight over that of GGT group. Nerve growth factor is a neurotrophic factor secreted by skeletal muscles. In this study, we applied the laser to the skin and muscle tissue; the areas we hypothesized contained increased levels of nerve growth factor. It has previously been shown that LLL therapy at a wavelength of 632 nm allows such a response [23]. LLL therapy appears to be an effective approach to accelerate the recovery of muscle fiber trophism. The results of this study demonstrate that LLL irradiation combined with the GGT nerve conduits can enhance peripheral nerve regeneration across a 15-mm gap in rats.

At twelve weeks after implantation, the GGT/LS group showed more compaction, continuity, and comprehensive histomorphology in regenerated nerve tissue compared with the GGT group. In addition, S-100 proteins are expressed mainly in Schwann cells, which are the principal glial cells of the peripheral nervous system. Schwann cells promote the growth, development, regeneration, and repair of nerves through the secretion of neurotrophic factors. Immunoreactivity to S-100 was also observed in the regenerated nerve segments of the GGT/LS group. The higher quantities of S-100 proteins suggest that GGT/LS treatments have greater potential to create a permissive environment for neuronal fiber growth inside the nerve grafts than the GGT treatment alone. 

In transverse sections of nerve conduits, the GGT/LS group also demonstrated greater neural tissue area, larger axon diameter, and thicker myelin sheaths than the GGT group, indicating better nerve regeneration. Myelin sheaths are an outgrowth of Schwann cells. Therefore, the superior recovery of myelin sheaths in the GGT/LS group could be considered evidence that laser stimulation activated Schwann cells. It has previously been shown that LLL enhances Schwann cell proliferation in vitro [24]. Schwann cells myelinate axons of the peripheral nervous system and play a crucial role in postinjury nerve regeneration. They promote neuronal survival, guide axons to their proper targets, and secrete neurotrophic factors that aid axonal elongation [25]. In our study, LLL therapy on injured nerves increased nerve function and improved the capacity for myelin production. Thus, the effectiveness of GaAlAsP diode laser (660 nm) with trigger point therapy for the promotion of axonal growth in injured nerves was demonstrated in an animal model.

Morphological changes in the mitochondria of lymphocytes, as well as the proliferation of mononuclear cells, were also observed after radiation with the red laser. These responses might be beneficial in the process of tissue repair [26, 27]. The underlying mechanism of phototherapy in nerve regeneration has been proposed in previous in vitro studies which showed that phototherapy induced Schwann cell proliferation [24], as well as massive neurite sprouting and outgrowth in cultured neuronal cells [28]. It has also been suggested that phototherapy may enhance the recovery of neurons by altering the oxidative metabolism of mitochondria [29]. The same mechanism may guide neuronal growth cones in vitro, perhaps through the interaction with cytoplasmic proteins and, in particular, by enhancing actin polymerization at the leading edge of the axon [30]. One possible molecular explanation is the increase in growth-associated protein-43 (GAP-43) immunoreactivity during the early stages of nerve regeneration proceeding phototherapy [31]. An additional effect of phototherapy on nervous tissue is a neuroprotective action which facilitates the regenerative capabilities of nerve fibers. It has been shown that phototherapy upregulates mRNA expression of calcitonin gene-related peptide (CGRP) in facial motor nuclei following axotomy. By increasing the intensity or altering the temporal pattern of injury-induced CGRP expression, phototherapy may optimize the rate of regeneration, target innervation and the survival of axotomized neurons [32]. In summary, all of the aforementioned effects may play a role in accelerating axonal regeneration and preventing the loss of neurons. Therefore, the rapid nerve regeneration induced by the GGT and LLL system may be a result of the synergistic effect produced by the unique properties of the GGT nerve conduit (permeability and structural stability), which provide an optimal environment for nerve regeneration, combined with the physical stimulus of LLL irradiation. Our study found that the GGT/LS treatment induces the following in injured peripheral nerves: (1) stimulation of Schwann cells to enhance neural tube formation; (2) activation of neurotrophic factors to accelerate axoplasm production; and (3) the incremental formation of new blood vessels to supply sufficient nutrients/oxygen.

## 5. Conclusion

This study used a previously developed biodegradable composite for use as a nerve conduit. The conduit was comprised of genipin-cross-linked gelatin annexed with *β*-tricalcium phosphate ceramic particles (GGT). The aim of this study was to investigate the influence of LLL trigger point therapy (using a 660 nm GaAlAsP diode laser) on the neurorehabilitation of transected sciatic nerves in rats after bridging them with the GGT nerve conduit. *In vivo* evaluation of rats indicates that the GGT nerve conduits successfully bridged a 15-mm gap in sciatic nerves and also possessed the required degradability to avoid an inflammatory response. Treating rats with LLL therapy improved motor functions, enhanced electrophysiological reactions, reduced muscle atrophy, and promoted histomorphometric recovery beyond that observed in the sham-irradiated rats. Quantitative results also show significantly greater changes in the morphometric values of regenerated nerves in the laser-treated group compared with those in the sham-irradiated group. These preliminary results support our hypothesis that LLL irradiation applied to transected nerves can enhance recovery. In conclusion, the present study investigated the use of LLL irradiation to stimulate growth of nerve segments in GGT nerve conduits and demonstrated that this biostimulus greatly improved peripheral nerve regeneration. These results indicate that combining the GGT nerve conduit with an LLL therapy system may be beneficial for the regeneration of nerves across long gaps, as well as for accelerating the reinnervation rate of nerves and improving recruitment in muscles. These benefits may in turn lead to improve functional and morphologic recovery of peripheral nerves. With regard to clinical applicability, this study makes an important contribution towards the development of a safe and effective strategy for rehabilitating peripheral nerve injuries. Further studies on the use of LLL therapy as a noninvasive treatment modality for various nerve diseases and injuries could pave the way for mainstream acceptance and standardization of this innovative therapy.

## Figures and Tables

**Table 1:** Recovery levels of sciatic nerve function in all rats during the experimental period.

Implantation time (week)	GGT Group	GGT/LS Group	*P *
Before surgery	−6.73 ± 3.82	−7.34 ± 3.11	0.79
1	−94.72 ± 2.51	−93.46 ± 1.34	0.36
2	−92.55 ± 2.33	−89.91 ± 2.97	0.16
4	−88.48 ± 3.15	−85.17 ± 3.05	0.18
6	−83.67 ± 1.48	−77.88 ± 2.26**	0.001
8	−78.34 ± 1.06	−73.25 ± 1.54**	0.001
10	−74.61 ± 1.42	−68.33 ± 1.21**	0.004
12	−72.83 ± 1.74	−66.14 ± 3.52**	0.004

Significantly **P < 0.01 greater than GGT group at the same implantation time.

**Table 2:** Morphometric parameters from the regenerated nerve tissue twelve weeks following implantation of GGT nerve conduits.

Morphometric parameters	GGT Group	GGT/LS Group	*P *
Medial nerve area (*μ*m^2^)	16,547 ± 544	20,163 ± 861***	<0.001
Nerve fiber diameter (*μ*m)	8.72 ± 0.26	10.31 ± 0.18***	<0.001
Nerve fiber number (0.1 mm^2^)	2,528 ± 314	3,322 ± 226**	0.002
Axon diameter (*μ*m)	4.13 ± 0.51	5.85 ± 0.33***	<0.001
Myelin sheath thickness (*μ*m)	2.26 ± 0.38	2.74 ± 0.11*	0.027

Significantly *P < 0.05, **P < 0.01, and ***P < 0.001 greater than GGT group.
